# Glances at the Hospitals

**Published:** 1901-09-14

**Authors:** 


### GLANCES AT THE HOSPITALS.

### WOOLWICH BRANCH OF THE METROPOLITAN PROVIDENT MEDICAL ASSOCIATION.

The seventh annual report of the Woolwich, Plumstead, and Charlton Branch of the Metropolitan Provident Medical Association shows that the general progress of the dispensary was well maintained during 1900, as in preceding years. In 1894 the number of members equalled 115, and their payments £13 10s. In 1900 the members had risen to 1,357, and they paid £279. The payments to doctors in the same period had increased from £34 to £148. During the year 1900 2,875 consultations were given by the medical officers at the dispensary or at their own surgeries, and 1,216 home visits were made to members. The total number of prescriptions dispensed was 5,694. Of the cases attended 21 were midwifery cases, 22 dentistry, 7 were sent to the Cottage Hospital, and others received treatment at the London hospitals, while several were sent to convalescent homes. Many members used the office of the dispensary as an information bureau with regard to the means of admission to the various hospitals. Surgical appliances were supplied, the money being banked by the members; and thanks are due to the district nurses who attended a number of patients in their own homes after confinement or surgical operations.

The plan of co-operation between the dispensary and the administration of the Hospital Saturday Fund, which enables many patients, particularly those suffering from chest complaints, to save journeys to London hospitals, continues to work with good results. During the year 80 patients presented examination forms at the dispensary and were examined by the medical officers: 22 were certified to be most suitable for treatment at a chest hospital, 5 were sent to a general hospital, and the remaining number were treated by local medical men. Of these, 48 obtained Hospital Saturday letters. In addition, 13 patients presented Hospital Saturday letters in the first instance and received advice and medicine at the dispensary. The medical staff gave in all 337 consultations.

A plan of co-operation with the Soldiers' and Sailors' Families' Association was successfully carried out, and 257 prescriptions were dispensed at a charge of 9d. each.

In November, in view of the fact that the Provident Dispensary is not yet large enough to be self-supporting, it was decided to raise a reserve fund of £100. Rather over half the sum was raised by the end of the year, and a donation enabled the committee to begin the new year with a clean sheet.

### THE BRADFORD EYE AND EAR HOSPITAL.

The total income was £2,454 and the expenditure was £2,835. The committee "regret to find that the annual subscriptions and other sources of income are not sufficient for the needs of the hospital." The annual subscriptions amounted to £840. The proportion of this contributed by the workpeople themselves is not given, but £278 was received from patients' payments, and as 6,126 cases were treated during 1899, this sum would average less than 1s. a head. Probably, therefore, this source of income could be greatly increased. We do not notice any statement relative to the cost per bed occupied, nor to the cost per head per week of the in-patients, nor to the cost per head of the out-patients. Of the total number treated during the year, 5,219 were eye cases, 907 were ear cases. The in-patients numbered 902 and the major operations were 970. The average stay of each patient in the hospital was nearly ten days. Extraction of the lens was performed 114 times for senile cataract, and 28 times for high myopia. In all cases the results are carefully tabulated. Anæsthetics were administered 354 times, chloroform being the agent used on 112 occasions, chloroform and ether mixture on 27 and nitrous oxide on 215.

### THE ROYAL INFIRMARY, SHEFFIELD.

The ordinary income of this hospital amounted to £10,423, and the ordinary expenditure to £14,381. The workmen's contributions amounted to the very respectable amount of £2,550. The cost of maintenance is not stated, so that we cannot form any opinion as to whether it is above or below the average of similar hospitals. At the beginning of the year there were 184 patients in the house, and 2,733 were admitted during the twelve months, making a total of 2,917. Of these 1,627 were discharged cured, 673 relieved, 82 as incurable, 64 for other reasons, 175 died, and 187 remained under treatment. There were 6,422 out-patients, and 14,244 "accidents and emergencies" were attended to. The medical and surgical tables are incomplete, inasmuch as they only state the number of patients under treatment for the various diseases. There is not much gained by knowing that 14 cases of appendicitis, 11 cases of enteric fever, and 54 cases of gastric ulcer were under treatment, if we hear nothing of the results of treatment. We notice that 775 surgical operations were performed, and this table is correctly drawn out, showing the results in all cases; but we venture to suggest an additional column for "remarks." In addition to the ordinary surgical operations there were 548 ophthalmic operations. In these the results are not given. There is no mention of the anæsthetic employed, save that five patients were examined under chloroform.

### THE SCARBOROUGH HOSPITAL AND DISPENSARY.

The total income reached £2,170 and the expenditure £2,380, showing a debit balance of £210. This was reduced to £60 by transferring £150 from the suspense account. The managers point out that the deficiency was in great part due to their having to renew their stock of linen and furniture, and to repairs to the road. The cost per bed occupied is not stated, nor is there any mention of the cost per week per patient. We are glad to note that the Board intend erecting a home for nurses on land adjoining the hospital; but they very wisely defer this work until the funds are actually in their hands. The total number of in-patients was 421, and the average stay in the hospital was 23 days. The medical and surgical tables set forth the various diseases under treatment, and separate columns show the numbers cured, relieved, not relieved, and died. The operations performed on the in-patients are all mentioned; but, oddly enough, the results are not given, and to learn these it would be essential to refer to the general tables, and even then we might be left in doubt. These tables show that 8 cases of hernia were under treatment and that all recovered, and the operations table shows that 6 of these were operated on, and therefore we infer that all the operations succeeded; but had there been a death mentioned in the general tables we should have been uncertain about the results of the operations. In the out-patients' department 4,085 patients were treated, and

409 in the eye and ear department. The dispensary statistics show that 5,376 patients were prescribed for, and that 1,291 of these were home patients, to whom the house surgeons made 8,355 visits; 1,484 cases were treated in the casualty department.

SUFFOLK GENERAL HOSPITAL, BURY ST. EDMUNDS.

The total income of this hospital amounted to £4,001 in 1899, and the total expenditure, including money invested, to £5,027. At the beginning of the year there were 45 patients in residence, and 570 were admitted during the year, making a total of 615. Of these 331 were cured, 35 relieved, 23 died, 181 were "renewed" or discharged for various reasons, and 45 remained under treatment. In the out-patients' department, 164 were under treatment on January 1st, and 1,649 were subsequently admitted. Of these 1,664 were discharged, and 149 remained at the end of the year. The average duration of each patient's stay in the hospital was 44 days. Seventy-four major operations were performed during the year. The medical and surgical details are extremely meagre, and convey no useful information.

BEDFORD COUNTY HOSPITAL.

The total income for the year 1899 was £4,585, and the total expenditure was £5,349, but the latter sum includes £947 for furniture for the new building. The average number of in-patients was 60; the average stay of each patient in the hospital was 29 days. The average weekly cost of provisions for each patient was 4s. 8¼d.; the average cost of each in-patient was £5 2s. 2d., and the average cost per bed occupied was £66 7s. 2d. The new hospital cost (including the nurses' home) £39,258, and £32,935 has been received, leaving a debt of £6,322 on the building fund. We regret this, as experience amply proves that these debts are not easily paid off, and have a tendency to cramp the usefulness of the hospital, for, of course, interest has to be paid on the debt. There were 51 patients in the hospital on January 1st, 1899; 715 were admitted since that date, 540 were discharged cured, 114 were relieved, 17 not relieved, 44 died, and 51 remained under treatment. The statistics given on pp. 71 and 72 gives the names of the various diseases and the numbers suffering from each disease, but no results are given. On the other hand, the operation table states all necessary details. We find that there were 16 cases of herniotomy, that all were cured, that ether was given in 4 cases and chloroform in 12. The total number of operations was 304. Of these 278 were successful, 14 partially so, 5 not successful, and 7 died. Ether was the anæsthetic employed in 39 cases and chloroform in 256.

THE GRAVESEND HOSPITAL.

At this hospital the income and expenditure almost balanced each other. Various collections amounted to £290, dividends to £350, donations to £1,149, patients' payments, private nursing receipts, &c., to £742, and subscriptions to £695. The hospital contained 39 patients at the beginning of the year, and as 480 were admitted during the year, the total number of in-patients was 519. Of these 279 were discharged cured, 147 relieved, 57 died, and 36 remained under treatment. There were 5,432 out-patients. The medical and surgical tables merely show the numbers of patients who suffered from the various diseases. Unless the results of treatment are given no useful object is served, and no comparison can be drawn. In like manner we see that 94 operations were performed, but we are left in doubt as to the success or failure of these operations. It is true that another table shows the causes of death in the 57 cases, but there is no ready means of identifying the one with the other. For instance, 10 cases of hernia were under treatment, 2 were operated on, and 1 died; but whether this was one of the latter or one of the former is left to our imagination. Neither do we see anything relative to the anæsthetic used in these 94 operations, nor is there any statement relative to the cost per patient, or to the weekly cost per head.

THE COTTAGE HOSPITAL AT FLEET, HANTS.

The balance remaining at the close of 1898 and the amount received during 1899 together amounted to £508. Of this sum £384 was expended, leaving £124 to the good; a condition of finance all too rare in connection with hospitals. In addition to the above mentioned ordinary income a sum of £316 was received from a bazaar, and this has been placed to the building account to meet the cost of the extensions now in progress. On January 1st, 1899, there were four patients in residence and 69 were admitted during the year. The average duration of treatment was about 26½ days. In a hospital of this size we do not look for extensive statistical tables, and we were agreeably disappointed in finding a list of patients treated during the year, the disease specified in each case, the date of admission and discharge given, and the result of the treatment stated. There were 36 operations performed, and among other cases it is stated that two patients suffering from tetanus were treated with Pasteur's anti-tetanic toxin and both recovered.

